# Usefulness of point-of-care multiplex PCR to rapidly identify pathogens responsible for ventilator-associated pneumonia and their resistance to antibiotics: an observational study

**DOI:** 10.1186/s13054-020-03102-2

**Published:** 2020-06-26

**Authors:** Charles-Edouard Luyt, Guillaume Hékimian, Isabelle Bonnet, Nicolas Bréchot, Matthieu Schmidt, Jérôme Robert, Alain Combes, Alexandra Aubry

**Affiliations:** 1 Service de Médecine Intensive Réanimation, Institut de Cardiologie, Hospital Pitié–Salpêtrière, Assistance Publique–Hôpitaux de Paris (APHP), Sorbonne Université, 47–83, Boulevard de l’Hôpital, 75651 Paris Cedex 13, France; 2 Sorbonne Université, INSERM, UMRS_1166-ICAN Institute of Cardiometabolism and Nutrition, Paris, France; 3 Service de Bactériologie–Hygiène, Hospital Pitié–Salpêtrière, APHP, Sorbonne Université, Paris, France; 4 Sorbonne Université, INSERM, U1135, Centre d’Immunologie et des Maladies Infectieuses, CIMI-Paris, équipe 2, Paris, France

**Keywords:** Ventilator-associated pneumonia, Antimicrobial stewardship, Multiplex PCR

## Abstract

**Background:**

The use of multiplex PCR to shorten time to identification of pathogens and their resistance mechanisms for patients with ventilator-associated pneumonia (VAP) is attractive, but poorly studied. The multiplex PCR–based Unyvero pneumonia cartridge assay can directly identify 20 bacteria and one fungus, amongst the most frequently causing VAP, and 19 of their resistance markers in clinical specimens (bronchoalveolar lavage or tracheal aspirate), with a turnaround time of 4–5 h. We performed this study to evaluate the concordance between the multiplex PCR–based Unyvero pneumonia cartridge assay and conventional microbiological techniques to identify pathogens and their resistance mechanisms in patients with VAP.

**Methods:**

All patients suspected of having VAP (January 2016 to January 2019), who underwent fiberoptic bronchoscopy with bronchoalveolar lavage fluid (BALF) and whose BALF microscopy examination revealed intracellular bacteria, were included. BALF conventional cultures (gold standard), antimicrobial susceptibility testing and processing for the Unyvero pneumonia cartridge were done. Culture and Unyvero results were compared.

**Results:**

Compared to cultures of the 93 samples processed for both techniques, Unyvero correctly identified pathogens in 68 (73%) proven VAP episodes, was discordant for 25 (27%), detected no pathogen in 11 and overdetected a not otherwise found pathogen in six. For the eight remaining discordant results, the pathogen responsible for VAP was not included in the Unyvero cartridge panel or it grew at a non-significant level in culture. Amongst the 31 (33%) resistance mechanism discordances observed, 22 were resistance detection failures and 24 concerned *Pseudomonas aeruginosa*.

**Conclusions:**

Compared to conventional microbiological cultures, the Unyvero pneumonia cartridge had poor diagnostic performance: it correctly identified pathogens and their resistance mechanisms in 73% and 67% of VAP cases, respectively. The lack of performance on the resistance mechanism was more pronounced when the pathogen detected was a *Pseudomonas aeruginosa*.

## Background

Rapid identification of pathogens responsible for ventilator-associated pneumonia (VAP) and their resistance mechanisms is a challenge in the intensive care unit (ICU). Indeed, conventional microbiological cultures (CMCs) require ≥ 48 h to grow the causative pathogens and determine their antimicrobial susceptibilities. While awaiting those results, empirical broad-spectrum antibiotics are prescribed [1–3]. A key issue in antimicrobial stewardship is decreasing consumption of broad-spectrum antibiotics [3]; shortening their empirical use may be a way to achieve that goal. Notably, molecular methods have been developed to supplement time-consuming CMCs, e.g. polymerase chain reaction (PCR) detection of bacterial DNA has been evaluated to shorten the time to diagnosis, but was restricted to specific pathogens and resistance mechanisms (e.g. *mecA* methicillin resistance in *Staphylococcus aureus* strains) [4]. Moreover, PCR is not available for pathogens usually causing VAP [5], or resistance mechanism identification requires a positive culture [4].

Recently, new multiplex PCRs (mPCRs) directly testing fresh samples have been developed to diagnose infections, including pneumonia. They target a panel of prevalent pneumonia-causing microorganisms, and some kits are commercially available. One of them, the mPCR Unyvero system pneumonia cartridge (Curetis GmbH, Holzgerlingen, Germany; henceforth Unyvero) can directly identify 20 bacteria and one fungus, amongst the most frequently causing VAP, and 19 of their resistance markers in clinical specimens, with a turnaround time of 4–5 h [6, 7]. Some published studies evaluated Unyvero, but their designs and test versions differed [6–11].

We undertook this prospective, observational study to evaluate the ability of the Unyvero pneumonia cartridge to diagnose VAP in ICU patients strongly suspected of being affected, i.e. with light microscopy visualisation of intracellular bacteria in bronchoalveolar lavage fluid (BALF).

## Methods

### Patients

Our ICU patients suspected of having VAP undergo fiberoptic bronchoscopy with BAL [8]. Half of BALF was sent to the bacteriology laboratory for CMM, and the rest was processed in ICU, as previously described [8, 9]: briefly, after cytocentrifugation and Diff-Quick staining, ICU physicians directly examine BALF by light microscopy for intracellular bacteria in neutrophils, thereby allowing 24/7 adaptation of empirical antimicrobials to the type of pathogens (i.e. bacilli, cocci or both) [8].

Between January 2016 and January 2019, all directly examined positive BALF (i.e. containing microscopy-detected intracellular bacteria) during office hours were included prospectively. VAP was diagnosed when all the following criteria were met: (1) clinically suspected VAP, defined as a new and persistent pulmonary infiltrate on chest radiograph associated with at least one of the following: temperature ≥ 38 °C, white blood cell (WBC) count ≥ 10 Giga/L and/or purulent tracheal secretions (for patients with acute respiratory distress syndrome, for whom demonstration of radiological deterioration was difficult, at least one of the three preceding criteria sufficed); (2) significant quantitative CMC growth (≥ 10^4^ cfu/mL) of distal BALF samples [12, 13].

### Gold standard: CMCs

The bacteriology laboratory processed BALF for CMCs. Antibiotic susceptibility was determined with the disc diffusion method, as recommended by the Antibiogram Committee of the French Society for Microbiology (CA-SFM), and the Alere® PBP2a rapid test (Abbott, Rungis, France) to identify methicillin resistance in *Staphylococcus aureus*.

### Unyvero P55 and HPN cartridges

Processing of BALF for the Unyvero P55 or the hospitalised pneumonia (HPN) cartridge was performed in the ICU following the manufacturer’s recommendations. At study onset, pneumonia cartridge P55 was used. During the study (September 2017), Curetis discontinued P55 and commercialised the upgraded HPN cartridge, incorporating *Chlamydophila pneumoniae* into the previous pathogen panel. The first 51 VAP episodes were tested with the P55 cartridge, and the remaining 42 with HPN.

Specimens were processed one or two at a time, either immediately after obtaining BALF or after storage at 4 °C for < 12 h, depending on the sampling time of the day. Briefly, 180 μL of the patient’s native sample underwent processing in a lysator for ~ 30 min; then, the lysis product and a master mix were loaded into a self-contained cartridge containing PCR primers (Curetis GmbH®) and placed in the analyser, where DNA was extracted, purified, amplified and specifically identified, generating complete diagnostic information within 4 h. To detect many analytes, eight mPCRs were run in parallel to detect panel-specific microarrays. The total time from obtaining BALF to results is a minimum of 4.5 h [6, 10]. The P55- and HPN-detected pathogens and resistance mechanisms are given in Supplementary Appendix Tables S1 and S2.

Unyvero results were collected and entered into the database but were not used to initiate or modify antimicrobial regimens.

### Data collection and analysis

The following data were prospectively collected: age, sex, Simplified Acute Physiology Score (SAPS) II, McCabe and Jackson Score for comorbidities, primary reason for ICU admission, date and reason for mechanical ventilation, antimicrobials received before VAP onset, clinical data at VAP onset and antimicrobial regimen for the VAP episode (including empirical and definite treatment).

CMCs served as the gold standard for the comparison between techniques, considering a test result: (1) a true positive, when CMC and Unyvero identified the same organism (CMC+, Unyvero+); (2) a false positive, when Unyvero but not CMC detected an organism (CMC−, Unyvero+); (3) a true negative, when neither method detected an organism (CMC−, Unyvero−); and (4) a false negative, when CMC but not Unyvero detected an organism (CMC+, Unyvero−). Sensitivity, specificity and positive and negative predictive values were calculated using those findings. The 95% confidence interval (95% CI) for test characteristics was calculated with Wilson’s method. We excluded resistance gene detection from this analysis because of too few data.

Data are expressed as median [interquartile range, IQR] or *n* (%). Analyses were computed using the StatView 5.0 (SAS Institute Inc., Cary, NC) software, with *p* < 0.05 defining significance.

### Ethics

In accordance with French law in January 2016 and our hospitals’ ethical committee recommendation (Committee for the Protection of Human Subjects Ile de France VI, Pitié-Salpêtrière Hospital), informed consent was not obtained because this observational study did not modify existing diagnostic or therapeutic strategies. However, patients and/or their relatives were informed about the anonymous data collection and were told that they could decline inclusion. This database is registered at the National Commission for Informatics and Liberties (CNIL registration no.: 1950673).

## Results

Ninety-three suspected VAP episodes in 83 patients were evaluated prospectively. Table 1 reports the baseline characteristics of the 83 patients, and Table 2 gives the characteristics of their 93 suspected VAP episodes.

**Table 1:** Baseline characteristics of 83 patients

Characteristic	Patients
Age, years	58 (43–64)
Male sex	63 (76)
Admission SAPS II	66 (52–76)
McCabe and Jackson Score for comorbidities ≥ 2	28 (34)
Primary reason for ICU admission	
Medical	61 (74)
Emergency surgery	20 (24)
Planned surgery	2 (2)
Reason for mechanical ventilation	
Shock	37 (45)
Acute respiratory failure	24 (29)
Postoperative respiratory failure	19 (23)
Coma	2 (2)
Others	1 (1)
Immunodepression	16 (19)
Chronic treatment with steroids	15 (18)
Risk factor for MDR bacteria	39 (47)
ICU mortality	42 (51)

Results are expressed as median (IQR) or *n* (%), as appropriate

*SAPS* Simplified Acute Physiology Score, *MDR* multidrug-resistant

**Table 2:** Clinical characteristics of the 93 suspected ventilator-associated pneumonia (VAP) episodes

Characteristic	Episodes
MV duration before VAP, days	9 (4–20)
Prior antimicrobial treatment	75 (81)
Broad-spectrum antimicrobials	52 (56)
Parameters at VAP onset	
Temperature, °C	37.2 (36.1–38.2)
White blood cell count, Giga/L	15.5 (10.3–23.1)
Neutrophil count, Giga/L	13.1 (8.1–19.1)
PaO_2_/FiO_2_ ratio, mmHg	130 (84–179)
mCPIS	5 (4–7)
Pathogen responsible for VAP^a^	
*Pseudomonas aeruginosa*	46 (49)
Other non-fermenting GNB	9 (10)
Enterobacteriaceae	53 (57)
*Escherichia coli*	17 (18)
*Enterobacter* spp.	1 (1)
*Klebsiella pneumoniae*	12 (13)
*Klebsiella oxytoca*	2 (2)
*Klebsiella variicola*	1 (1)
*Proteus mirabilis*	6 (6)
*Morganella morganii*	1 (1)
*Serratia marcescens*	1 (1)
*Citrobacter freundii*	1 (1)
*Proteus vulgaris*	1 (1)
*Staphylococcus aureus*	4 (4)
*Haemophilus influenzae*	3 (3)
*Enterococcus* spp.	2 (2)
Polymicrobial oropharyngeal flora	3 (3)
Miscellaneous^b^	3 (3)
Negative BAL	2 (2)
Positive blood culture	5 (5)
Days of antimicrobial treatment	8 (6–8)

Results are expressed as median (IQR) or *n* (%)

*MV* mechanical ventilation, *VAP* ventilator-associated pneumonia, *mCPIS* Modified Clinical Pulmonary Infection Score [14], *BALF* bronchoalveolar lavage fluid

^a^According to conventional microbiological cultures; the total number of pathogens exceeds 93 because 27 patients had at least two pathogens responsible for VAP

^b^*Achromobacter xylosoxidans*, *Kluyvera ascorbata* or *Raoultella ornithinolytica*, one each

### CMC and Unyvero concordance

#### Pathogen identification

Amongst the 93 suspected VAP episodes, Unyvero agreed with CMCs for 68 (73%) of them and differed for 25 (27%). Unyvero correctly detected the pathogens in two episodes, but their growth was non-significant (< 10^4^ cfu/mL). These discordances are detailed in Table 3.

**Table 3:** The 25 pathogen detection discordances between conventional microbiological cultures and Unyvero

Discordance	Conventional cultures^a^	Unyvero*
Unyvero failed to detect a pathogen		
1	***Klebsiella pneumoniae***	
	*Escherichia coli*	*Escherichia coli*
2	***Klebsiella pneumoniae***	*Stenotrophomonas maltophilia*
	*Stenotrophomonas maltophilia*	
3^b^	***Enterobacter aerogenes***	***Klebsiella oxytoca***
	***Proteus mirabilis***	
4	***Staphylococcus aureus***	
	*Acinetobacter baumannii*	*Acinetobacter baumannii*
5	***Klebsiella pneumoniae***	
	*Escherichia coli*	*Escherichia coli*
	*Morganella morganii*	*Morganella morganii*
6	***Enterobacter aerogenes***	
	*Klebsiella pneumoniae*	*Klebsiella pneumoniae*
7	***Enterobacter aerogenes***	–
8	***Klebsiella oxytoca***	–
9	***Klebsiella pneumoniae***	–
10	***Staphylococcus aureus***	–
11	***Klebsiella variicola***	–
Conventional cultures detected no specific pathogen		
12	**Oropharyngeal flora**	–
13	**Oropharyngeal flora**	–
14	**Oropharyngeal flora**	–
Pathogen not in the Unyvero panel		
15	***Kluyvera ascorbata***	*Enterobacter cloacae*
16	***Raoultella ornithinolytica***	
	*Proteus mirabilis*	*Proteus* spp.
17	***Achromobacter xylosoxidans***	
	*Stenotrophomonas maltophilia*	*Stenotrophomonas maltophilia*
18	***Enterococcus faecium***	*–*
19	***Enterococcus faecium***	
	*Stenotrophomonas maltophilia*	*Stenotrophomonas maltophilia*
Unyvero overdetected pathogens		
20	*Pseudomonas aeruginosa*	*Pseudomonas aeruginosa*
	*Serratia marcescens*	*Serratia marcescens*
	*Klebsiella pneumoniae*	*Klebsiella pneumoniae*
		***Escherichia coli***
21	*Enterobacter cloacae*	*Enterobacter cloacae*
		***Stenotrophomonas maltophilia***
22	*Pseudomonas aeruginosa*	*Pseudomonas aeruginosa*
		***Stenotrophomonas maltophilia***
23	*–*	***Staphylococcus aureus***
24	*Pseudomonas aeruginosa*	*Pseudomonas aeruginosa*
		***Stenotrophomonas maltophilia***
25	*Pseudomonas aeruginosa*	*Pseudomonas aeruginosa*
		***Stenotrophomonas maltophilia***

^a^False results (positive or negative) are in bold type

^b^This episode had false-negative and false-positive findings

Discordance patterns were classified as false positive for six of the 25 episodes and false negative for the other 19. Amongst the latter, discordance patterns varied. A VAP causative pathogen was not included in the Unyvero panel for five episodes (*Enterococcus faecium* for two; *Achromobacter xylosoxidans*, *Kluyvera ascorbata* or *Raoultella ornithinolytica* for one each). Unyvero failed to detect a pathogen for 11 episodes, despite their significant growth levels (> 10^4^ cfu/mL): for five Unyvero− results, a pathogen was retrieved from CMCs; the two methods detected the same pathogens for six episodes, but CMCs grew a second pathogen not detected by Unyvero. For three episodes, CMCs grew “oropharyngeal flora” with no single causative pathogen, with Unyvero− for all three.

Unyvero yielded six false-positive results for *Stenotrophomonas maltophilia*, in addition to other bacteria in four episodes; *S. aureus* in one episode that was CMC−; and *Escherichia coli* in addition to other bacteria in the last episode.

Compared to CMCs, Unyvero had 77.4% sensitivity and 14.3% specificity for pathogen identification (Table 4). When analysed separately, P55 and HPN cartridge results did not differ. Moreover, the analysis yielded similar concordance rates of patients with or without previous antimicrobial treatment (Table 4).

**Table 4:** Comparisons of Unyvero vs conventional microbiological methods for all episodes, according to Unyvero version and to previous antibiotic use

Finding	*N* positive		*N* negative		Sensitivity	Specificity (%)	PPV (%)	NPV (%)
	True	False	True	False				
Total (*n* = 93)								
Pathogen identification	65	6	1	19	77.4	14.3	91.5	5
Resistance mechanism	19	9	43	22	46.3	82.7	67.9	66.2
P55 cartridge (*n* = 51)								
Pathogen identification	39	1	1	10	79.6	50	97.5	9
Resistance mechanism	12	7	21	11	52.1	75	63.2	65.6
HPN cartridge (*n* = 42)								
Pathogen identification	26	5	0	9	74.3	0	83.9	0
Resistance mechanism	7	2	22	11	38.9	91.7	77.8	66.7
Previous antimicrobial treatment (*n* = 75)								
Pathogen identification	54	6	1	14	79.4	14.2	90	6.7
Resistance mechanism	15	8	31	21	41.7	79.5	65.2	59.6
No previous antimicrobial treatment (*n* = 18)								
Pathogen identification	13	0	0	5	72.2	0	100	0
Resistance mechanism	4	1	12	1	80	92.3	80	92.3

Conventional microbiological methods were considered as the gold standard

*PPV* positive predictive value, *NPV* negative predictive value, *HPN* hospitalised pneumonia

#### Antimicrobial resistance detection

Resistance mechanisms were in concordance for 62 episodes (Table 4), while Unyvero and CMCs differed for 31 (33%) episodes, mostly when *Pseudomonas aeruginosa* recovered (*n* = 24, 77%). Moreover, for most episodes, discordance was primarily attributable to Unyvero’s failure to detect resistance (71%).

Discordance patterns for *P. aeruginosa* were as follows: false resistance to fluoroquinolones for seven episodes and carbapenem or third-generation cephalosporin resistance not found in five and 12 episodes, respectively.

Excluding *P. aeruginosa*, seven resistance mechanism discordances were observed between Unyvero and CMCs: false resistance to fluoroquinolones for two episodes (*E. coli* recovered from both), penicillin resistance not detected in one (*Proteus mirabilis-*infected patient) and third-generation cephalosporin not identified in four episodes (Unyvero failed to detect extended-spectrum beta-lactamase (ESBL)–producing *Klebsiella pneumoniae*, with Unyvero accurately detecting ESBL-carrying causative *E. coli* in two episodes or *K. pneumoniae* in one, but not its resistance mechanism). For the latter four episodes, the patients’ ESBL-producing Enterobacteriaceae colonisation was known.

## Discussion

Herein, the Unyvero point-of-care tool correctly detected the VAP-causing pathogen for 73% of the episodes and identified the correct resistance mechanism in 67% of them. Intriguingly, the resistance mechanism discordance rate differed when *P. aeruginosa* was the causative agent, compared to other microorganisms.

Rapid detection of the pathogens responsible for VAP and their resistant mechanisms is a critical issue for ICU patients. To date, six published studies investigated the usefulness of the mPCR-based Unyvero to achieve this goal; their concordance results between the cartridge and CMCs were heterogeneous (Table 5) [6–11]. However, most had used the older test version; only Gadsby et al. [13] evaluated the P55 cartridge, like us, and we were the only ones to assess the most recent HPN cartridge. Results differed across studies, with recent versions better identifying the pathogens. The four studies that examined CMC–Unyvero resistance concordance found similar rates of ~ 70% (Table 5).

**Table 5:** Studies that evaluated the usefulness of the Unyvero pneumonia cartridge for patients suspected of having lower respiratory tract infections

Author, year [ref]	Study design	Population	Test	Specimen type	*N* specimens/*N* patients	Pathogen identification		Concordant resistance identification
						Concordance*	Se/Sp	
Schulte, 2014 [8]	Prospective observational, fresh samples	Adults with suspected HAP/VAP	Unyvero P50	BALF, ETA, sputum	739/NR	NR	70.6%/95.2%	–
Jamal, 2014 [6]	Prospective observational, fresh samples	Children and adults with suspected HAP/VAP	Unyvero P50	BALF, ETA, sputum	49/49	23/49 (47%)	NR	NR
Kunze, 2015 [7]	Prospective observational, fresh samples	Adults with suspected HAP	Unyvero P50	ETA, NPTA	40/40	18/40 (45%)	75%/43%	–
Personne, 2016 [9]	Prospective observational, fresh samples	Adults with suspected pneumonia	Unyvero P50	NR	90/NR	59/90 (66%)	95.7%/32.6%	75.6%
Papan, 2018 [10]	Prospective observational, fresh samples	Children and neonates with suspected pneumonia	Unyvero P50	BALF, ETA, pleural fluid	79/79	48/80 (60%)	73.1%/97.8%	75%
Gadsby, 2019 [11]	Prospective observational, frozen samples	Adults with suspected VAP, CAP or HAP	Unyvero P55	BALF	74/74	57/99 (57.5%)	56.9%/58.5%	121/166 (72.9%)
This study	Prospective observational, fresh samples	Adults with suspected VAP and bacteria in BALF	Unyvero P55 and HPN	BALF	93/83	71%	77.4%/17.3%	62/93 (67%)

*Se/Sp* sensitivity/specificity, *BALF* bronchoalveolar lavage fluid, *ETA* endotracheal aspirate, *NPTA* nasopharyngeal tracheal aspirate, *NR* not reported, *HAP* hospital-acquired pneumonia, *VAP* ventilator-associated pneumonia, *CAP* community-acquired pneumonia, *HPN* hospitalised pneumonia

*Both concordant positive (correct pathogen identification by both methods) and concordant negative (no pathogen identification by both methods)

Our study differs from the others in several ways. First, we exclusively studied patients with suspected VAP, not hospital-acquired or community-acquired pneumonia. Second, we used the most recent version (but switched from the discontinued P55 to HPN cartridges during the study). Third, we focused on patients strongly suspected of having VAP, primarily to avoid expenditures for unnecessary tests in a context of low pretest pneumonia probability and also to assess Unyvero’s usefulness within our care organisation. These latter points could explain the very low specificity observed.

Our results showed the usual limitations of PCR-based tools for detecting pathogens in respiratory samples. The first is the overdetection (false positive), i.e. pathogen detection without pneumonia, seen in six episodes. Such overdetection may indicate nucleic acids of non-viable organisms or bacterial presence without reaching a pathogenic threshold (colonisation). One of the advantages of Unyvero’s P55 and HPN cartridges is their semi-quantification, with results being positive only when the sample’s bacterial burden is sufficiently high. Unfortunately, this system does not allow having a true quantification of the bacterial burden. The second limitation of this kind of test is underdetection (false negative), i.e. no pathogen detected despite significant pathogen growth in CMCs, as observed for 19 episodes. One explanation for underdetection was the absence of the VAP causative pathogen in the test panel.

Notably, resistance mechanism discordances were more frequent when *P. aeruginosa*, rather than another microorganism, was the responsible agent, perhaps explained *P. aeruginosa*’s changing resistance profile over time and that the PCR recognises only 17 resistance markers against Gram-negative bacilli (mostly carbapenem resistance; Supplementary Appendix Table S2) [15, 16]. We observed that Unyvero did not identify ESBL in four episodes of ESBL-producing Enterobacteriaceae VAP. Although ESBL enzymes which were present in those patients are unknown (Supplementary Appendix Table S2), Unyvero does not include all ESBL in its assay panel. Importantly, all four patients with false-negative results were known to be colonised by ESBL-producing Enterobacteriaceae. Therefore, our results do not support the current routine use of this system to adapt antimicrobial treatment.

Several limitations should be underlined. First, this was a monocentric, prospective study, and despite having included a large number of patients, our results warrant further investigations. Second, the strategy chosen required fiberoptic bronchoscopy and BAL, which are not universally available and remain debated as a first-line tool for diagnosing VAP [1, 2]. However, using tracheal aspirates or sputum may generate more false positives with Unyvero. Third, although Unyvero targets the most frequent pathogens responsible for hospital-acquired pneumonia, some VAP causative microorganisms are missing from the cartridge panel. Moreover, polymicrobial VAP (e.g. oropharyngeal flora) may be missed using this system. However, to overcome these limitations, we propose an algorithm based first on direct BALF examination, then Unyvero if bacilli are found. Fourth, all samples were not processed with the same cartridge, since the manufacturer shifted from P55 to HPN during our study. Nonetheless, the two cartridges differ only by the addition of *Chlamydophila pneumoniae* to the HPN cartridge; because this pathogen is not frequently responsible for VAP and was never detected in our patients, the results would have not been different if we had used the same cartridge throughout the study. Indeed, results were similar when P55 and HPN cartridges were compared. Lastly, molecular tests were not run to explore discordances between the two techniques and to characterise resistance mechanisms.

## Conclusions

In conclusion, the Unyvero pneumonia cartridges correctly detected VAP causative pathogens for 73% of the episodes and correctly identified the resistance mechanism in 67% of them, differing according to the responsible pathogen, with *P. aeruginosa* having the highest discordance rate.

## Supplementary information

**Additional file 1: Supplementary Table S1.** Pathogens detected by the Unyvero hospitalised-pneumonia (HPN) cartridge **Supplementary Table S2.** Resistance markers potentially detected by the HPN system and their target(s).

## Data Availability

The datasets generated during the current study are available from the corresponding author on reasonable request.

## Abbreviation

BALF: Bronchoalveolar lavage fluid
CMC: Conventional microbiological cultures
DNA: Deoxyribonucleic acid
ESBL: Extended-spectrum beta-lactamase
HPN: Hospitalised pneumonia
ICU: Intensive care unit
IQR: Interquartile range
PCR: Polymerase chain reaction
SAPS: Simplified Acute Physiology Score
VAP: Ventilator-associated pneumonia
WBC: White blood cell

## References

[1] Kalil AC, Metersky ML, Klompas M, Muscedere J, Sweeney DA, Palmer LB (2016). Management of adults with hospital-acquired and ventilator-associated pneumonia: 2016 Clinical Practice Guidelines by the Infectious Diseases Society of America and the American Thoracic Society. Clin Infect Dis.

[2] Torres A, Niederman MS, Chastre J, Ewig S, Fernandez-Vandellos P, Hanberger H, et al. International ERS/ESICM/ESCMID/ALAT guidelines for the management of hospital-acquired pneumonia and ventilator-associated pneumonia: guidelines for the management of hospital-acquired pneumonia (HAP)/ventilator-associated pneumonia (VAP) of the European Respiratory Society (ERS), European Society of Intensive Care Medicine (ESICM), European Society of Clinical Microbiology and Infectious Diseases (ESCMID) and Asociación Latinoamericana del Tórax (ALAT). Eur Respir J. 2017;50:1700582. 10.1183/13993003.00582-2017 28890434

[3] Luyt C-E, Bréchot N, Trouillet J-L, Chastre J (2014). Antibiotic stewardship in the intensive care unit. Crit Care.

[4] Thomas LC, Gidding HF, Ginn AN, Olma T, Iredell J (2007). Development of a real-time Staphylococcus aureus and MRSA (SAM-) PCR for routine blood culture. J Microbiol Methods.

[5] Fullston EF, Doyle MJ, Higgins MF, Knowles SJ. Clinical impact of rapid polymerase chain reaction (PCR) test for group B Streptococcus (GBS) in term women with ruptured membranes. Ir J Med Sci. 2019;188:1269–74. 10.1007/s11845-019-01977-x 30706295

[6] Jamal W, Al Roomi E, AbdulAziz LR, Rotimi VO (2014). Evaluation of Curetis Unyvero, a multiplex PCR-based testing system, for rapid detection of bacteria and antibiotic resistance and impact of the assay on management of severe nosocomial pneumonia. J Clin Microbiol.

[7] Kunze N, Moerer O, Steinmetz N, Schulze MH, Quintel M, Perl T (2015). Point-of-care multiplex PCR promises short turnaround times for microbial testing in hospital-acquired pneumonia--an observational pilot study in critical ill patients. Ann Clin Microbiol Antimicrob.

[8] Schulte B, Eickmeyer H, Heininger A, Juretzek S, Karrasch M, Denis O (2014). Detection of pneumonia associated pathogens using a prototype multiplexed pneumonia test in hospitalized patients with severe pneumonia. PLoS One.

[9] Personne Y, Ozongwu C, Platt G, Basurto-Lozada P, Shamin M, Gant VA (2016). “Sample-in, answer-out”? Evaluation and comprehensive analysis of the Unyvero P50 pneumonia assay. Diagn Microbiol Infect Dis.

[10] Papan C, Meyer-Buehn M, Laniado G, Nicolai T, Griese M, Huebner J (2018). Assessment of the multiplex PCR-based assay Unyvero pneumonia application for detection of bacterial pathogens and antibiotic resistance genes in children and neonates. Infection..

[11] Gadsby NJ, McHugh MP, Forbes C, MacKenzie L, Hamilton SKD, Griffith DM (2019). Comparison of Unyvero P55 pneumonia cartridge, in-house PCR and culture for the identification of respiratory pathogens and antibiotic resistance in bronchoalveolar lavage fluids in the critical care setting. Eur J Clin Microbiol Infect Dis.

[12] Chastre J, Fagon J-Y (2002). Ventilator-associated pneumonia. Am J Respir Crit Care Med.

[13] Chastre J, Luyt C-E (2016). Does this patient have VAP?. Intensive Care Med.

[14] Luyt C-E, Chastre J, Fagon J-Y (2004). Value of the clinical pulmonary infection score for the identification and management of ventilator-associated pneumonia. Intensive Care Med.

[15] El Zowalaty ME, Al Thani AA, Webster TJ, El Zowalaty AE, Schweizer HP, Nasrallah GK (2015). Pseudomonas aeruginosa: arsenal of resistance mechanisms, decades of changing resistance profiles, and future antimicrobial therapies. Future Microbiol.

[16] Eichenberger EM, Thaden JT. Epidemiology and mechanisms of resistance of extensively drug resistant Gram-negative bacteria. Antibiotics (Basel). 2019;8:37. 10.3390/antibiotics8020037 PMC6628318 30959901

