# Supplementary material for: Usefulness of point-of-care multiplex PCR to rapidly identify pathogens responsible for ventilator-associated pneumonia and their resistance to antibiotics: an observational study
Source: Crit Care. 2020 Jun 26;24:378. doi: 10.1186/s13054-020-03102-2 (PMC7316635; doi:10.1186/s13054-020-03102-2)
Supplement: Supplementary file 1 — Additional file 1: Supplementary Table S1. Pathogens detected by the Unyvero hospitalised-pneumonia (HPN) cartridge Supplementary Table S2. Resistance markers potentially detected by the HPN system and their target(s). [file 13054_2020_3102_MOESM1_ESM.docx]

**Usefulness of point-of-care multiplex PCR for rapid identification of pathogens responsible for ventilator-associated pneumonia**

CE Luyt, G Hékimian, I Bonnet, N Bréchot, M Schmidt, J Robert, A Combes, A Aubry

Online supplement

**Supplementary Table S1**

Pathogens detected by the Unyvero hospitalised-pneumonia (HPN) cartridge

| Pathogens detected |
| --- |
| Gram-positive |
| *Staphylococcus aureus* |
| *Streptococcus pneumoniae* |
| Enterobacteriaceae |
| *Escherichia coli* |
| *Enterobacter cloacae* complex |
| *Enterobacter aerogenes* |
| *Proteus* spp*.* |
| *Morganella morganii* |
| *Serratia marcescens* |
| *Citrobacter freundii* |
| *Klebsiella pneumoniae* |
| *Klebsiella oxytoca* |
| *Klebsiella variicola* |
| Non-fermenting bacteria |
| *Pseudomonas aeruginosa* |
| *Acinetobacter baumannii* complex |
| *Legionella pneumophila* |
| *Moraxella catarrhalis* |
| *Stenotrophomonas maltophilia* |
| Miscellaneous |
| *Haemophilus influenzae* |
| *Chlamydophila pneumoniae* |
| *Pneumocystis jirovecii* |
| *Mycoplasma pneumoniae* |

The P55 cartridge, used for the first 51 patients included, targeted the same pathogens except *Chlamydophila pneumonia*, which was added to the HPN-cartridge panel.

**Supplementary Table S2**

Resistance markers potentially detected by the HPN system and their target(s)

| **Resistance gene or chromosomal mutation** | **Resistance to** | **Pathogen(s) targeted** |
| --- | --- | --- |
| *mecA* | Oxacillin | *Staphylococcus* spp. |
| *mecC* | Oxacillin | *Staphylococcus* spp. |
| *ermB* | Macrolides/lincosamides | *Streptococcus* spp. |
| *Tem* | Penicillin | Enterobacteriaceae, non-fermenting GNB, *Haemophilus influenzae* |
| *SHV* | Penicillin | Enterobacteriaceae, non-fermenting GNB |
| *Tem+SHV* | Third-generation cephalosporins | Enterobacteriaceae, non-fermenting GNB |
| *Oxa-23* | Carbapenems | Enterobacteriaceae, non-fermenting GNB |
| *Oxa-24* | Carbapenems | Enterobacteriaceae, non-fermenting GNB |
| *Oxa-48* | Carbapenems | Enterobacteriaceae, non-fermenting GNB |
| *Oxa-58* | Carbapenems | Enterobacteriaceae, non-fermenting GNB |
| *Vim* | Carbapenems | Enterobacteriaceae, non-fermenting GNB |
| *Imp* | Carbapenems | Enterobacteriaceae, non-fermenting GNB |
| *KPC* | Carbapenems | Enterobacteriaceae, non-fermenting GNB |
| *NDM* | Carbapenems | Enterobacteriaceae, non-fermenting GNB |
| *Ctx-M* | Third-generation cephalosporins | Enterobacteriaceae, non-fermenting GNB |
| *Sul1* | Sulfonamides | Enterobacteriaceae, non-fermenting GNB |
| *gyrA8*3_Ecoli | Fluoroquinolones | *Escherichia coli* |
| *gyrA87*_Ecoli | Fluoroquinolones | *Escherichia coli* |
| *gyrA83*_Pseu | Fluoroquinolones | *Pseudomonas aeruginosa* |
| *gyrA87*_Ecoli | Fluoroquinolones | *Pseudomonas aeruginosa* |
